# Effect Of Microgravity On Aromatase Expression In Sertoli Cells

**DOI:** 10.1038/s41598-017-02018-2

**Published:** 2017-06-14

**Authors:** Elisa Cirelli, Emanuela De Domenico, Flavia Botti, Renato Massoud, Raffaele Geremia, Paola Grimaldi

**Affiliations:** 1 Department of Biomedicine and Prevention, University of Rome Tor Vergata, 00133 Rome, Italy; 2 Department of Clinical Sciences and Translational Medicine, University of Rome Tor Vergata, 00133 Rome, Italy; 3 Department of Experimental Medicine and Surgery, University of Rome Tor Vergata, 00133 Rome, Italy

## Abstract

Cytochrome P450-aromatase catalyzes estrogen biosynthesis from C_19_ steroids. In the testis, Sertoli cells express P450-aromatase and represent the primary source of estrogen during prepuberal age. This study focused on the effect of simulated microgravity (SM) on aromatase expression in primary mouse Sertoli cells. When cultured in Rotary Cell Culture System (RCCS), Sertoli cells, formed multicellular three dimensional spheroids (3D). Biological properties were first analyzed in terms of viability, cell cycle, expression of cytoskeletal components and growth factors in comparison to Sertoli cells cultured in spheroids at unit gravity (G). SM did not affect cell viability and proliferation, nor expression of the main cytoskeleton proteins and of growth factors like Kit Ligand (KL) and glial derived neurotrophic factor (GDNF). On the other hand, SM caused a strong increase in P450 aromatase mRNA and protein expression. Interestingly, P450-aromatase was no more inducible by 8-Br-cAMP. The presence of a functional aromatase was confirmed by enrichment of 17β-estradiol released in the medium by androgen precursors. We concluded that SM causes a significant upregulation of aromatase gene expression in Sertoli cells, leading to a consequent increase in 17β-estradiol secretion. High level of 17β-estradiol in the testis could have potentially adverse effects on male fertility and testicular cancer.

## Introduction

Spermatogenesis is a complex process regulated by gonadotropins and steroid hormones and modulated by a network of autocrine and paracrine factors^1^. These modulators ensure the correct progression of germ cell differentiation and the production of mature spermatozoa. Their expression and function can be affected by environmental conditions. In this regard, some adverse effects on male reproduction in humans and other mammals have been observed during space flights and in ground-based experiments. These studies have demonstrated that microgravity results in alteration of spermatogenesis^2^, of the integrity of the blood-testis barrier^3^ and in changes in hormone levels^4^, such as testosterone (T), follicle stimulating hormone (FSH) and luteinizing hormone (LH). Low levels of testosterone have been detected in humans and rat during space flights^5, 6^. One of the most important factors that affects testosterone levels is the activity of the P450-aromatase enzyme, which converts testosterone to estrogen, thus depleting free testosterone and increasing estrogen levels. The P450-aromatase enzyme is encoded by the CYP19A gene^7^ and is expressed in all testicular cells except peritubular cells^8^. Male mice deficient in P450-aromatase are initially fertile but show disrupted spermatogenesis and infertility upon aging^9^. Moreover, overexpression of the P450-aromatase gene and the enhanced 17β-estradiol (E2) production in mice induced cryptorchidism or undescended testis and spermatogenic arrest, leading to male infertility in all animals when it takes place in fetal life, or in 50% of them when it occurs at puberty^10^. P450-aromatase enzyme is present in fetal and neonatal Sertoli cells and its expression is downregulated during maturation^11^, while in the adult rat testis, it is expressed mainly in Leydig cells and in germ cells^12–14^. The role of estrogens produced by immature Sertoli cells is not well understood, but it is remarkable that knockout of the genes for either aromatase or estrogen receptors can result in the latent appearance of ‘Sertoli-like’ cells in the ovaries of females^15, 16^, suggesting a role for estrogens in Sertoli cell differentiation. Indeed it has been reported a significant role for estrogen in establishing Sertoli cell function^17^ and Sertoli-germ cell adhesion in the developing testis^18^. Herein, we focused on isolated Sertoli cell populations, and we established and characterized a three dimensional (3D) cell culture system in RCCS to study the primary effects of gravitational changes on the expression of aromatase at mRNA and protein level in these cells.

## Results

### Sertoli cells form spheroids in RCCS

**Figure 1:**
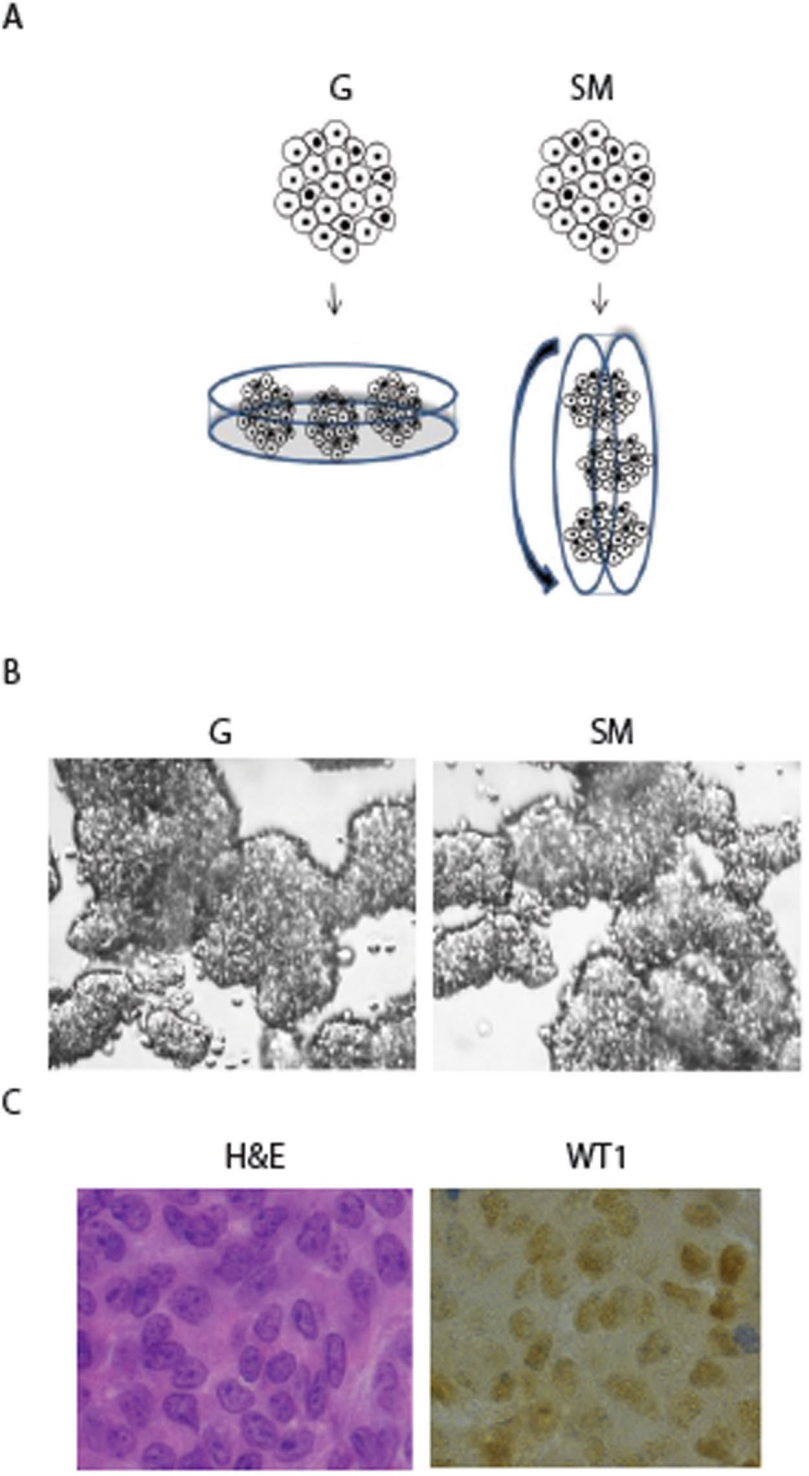
SM induces Sertoli cell spheroids. (**A**) Schematic representation of 3D Sertoli cell culture at unit gravity (G) and in Rotary Cell Culture System (SM). (**B**) Representative images of Sertoli cell spheroids after 48 hours of culture at G or under SM. (**C**) Representative sections of Sertoli cell spheroids immunostained with WT1 antiboby, a marker of Sertoli cells and stained with H&E.

To investigate the effects of microgravity on Sertoli cells, we used the Rotary Cell Culture System (RCCS), a microgravity based bioreactor^19, 20^. Under this condition, mouse Sertoli cells obtained from 17-days-old mice showed a round-shaped phenotype and aggregated into three dimensional (3D) multicellular spheroids (Fig. 1A). Sertoli cell cultured in RCCS were compared with 3D Sertoli cell aggregates cultured at unit gravity (G). They were obtained by plating the cells on plastic dishes pretreated with soft agar that, not allowing cell adhesion, forced the cells to grow in suspension and to form cell aggregates, similar in shape and size to those grown in RCCS (Fig. 1A,B). The cell-spheroids formed in RCCS or at G were composed almost exclusively of Sertoli cells, as revealed by immunohistochemistry with anti-WT1 antibody, a Sertoli cell marker, indicating a very high purity of cell population (Fig. 1C).

**Figure 2:**
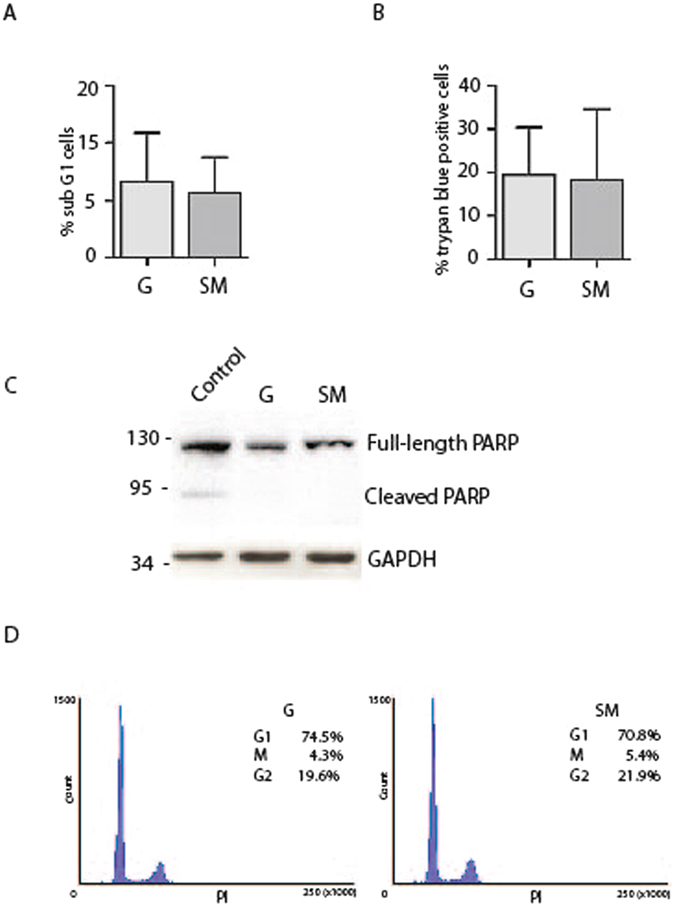
SM does not cause significative changes in Sertoli cell viability and cell cycle. (**A**) Percentage of sub G1 Sertoli cells cultured at G or under SM. Sertoli were cultured for 48 h and then analyzed by FACS after propidium iodide staining. (**B**) Percentage of Trypan blue positive Sertoli cells detected after 48 h of culture at G or under SM. (**C**) Analysis of cleaved-PARP in protein extracts from Sertoli cells cultured at G or under SM showing the absence of cleaved forms in SM and G conditions. In Control lane is reported a positive control in which embryonal carcinoma cells (2102EP cell line) were treated for 24 h with 3.3 µM cisplatin. A cleaved band at around 89 kDa is shown. (**D**) Cell cycle analysis of Sertoli cells cultured for 48 h at G or under SM and then analyzed by FACS after propidium iodide staining. Most of the cells are in G1 phase in both culture conditions. At least three different experiments were performed. Bars represent s.d.

The effect of microgravity on cell survival was investigated after 48 hours of culture in RCCS by FACS (Fluorescence Activated Cell Sorter) analysis of cell cycle and by Trypan blue staining. As reported in Fig. 2A the percentage of cells in sub G1, which represented the apoptotic cells, was very low and did not change between RCCS and G conditions (Fig. 2A). Similarly, no significant modifications in the percentage of Trypan blue positive cells could be detected (Fig. 2B), indicating that microgravity did not affect cell survival. We also analyzed the expression of PARP-1 protein as marker of apoptosis, whose cleavage in fragments of 89 and 24 kDa represents a useful hallmark of this type of cell death. We showed that cleaved PARP-1 fragments were not detectable in 3D cultures neither at G or in RCCS (Fig. 2C) confirming that Sertoli cells viability is not affected by the culture conditions used. A band corresponding to 89kDa- cleaved PARP-1 was detected in embryonal carcinoma cell line treated with cisplatin used as positive control.

During perinatal period, Sertoli cells proliferate quickly and, at around the onset of puberty, they switch from an immature, proliferative state to a mature, non-proliferative state. In mouse, immature Sertoli cells are proliferative until 10–13dpp (days post partum), at which point they permanently exit the cell cycle and are considered a stable and terminally differentiated population. However, since evidence from the literature is now challenging this dogma and it has been reported that Sertoli cells can resume proliferation^21^, we set out to investigate Sertoli cell proliferation in our cultures by FACS analysis. Sertoli cells cultured in spheroids in RCCS were mostly found in G1 phase (79.8% ± 6.4%) and no significant difference was detected between them and the cells at G (74.5% ± 7.5%) (Fig. 2D). This result suggests that Sertoli cells from 17dpp mice were in a quiescent state and SM did not influence their cell cycle progression. A similar conclusion was reached by cell cycle analysis after incorporation of BrdU, which did not give any staining of cells (data not shown). Our results indicate that SM did not induce significant alterations in viability and cell cycle progression in Sertoli cells.

### Sertoli cell biological properties in RCCS

**Figure 3:**
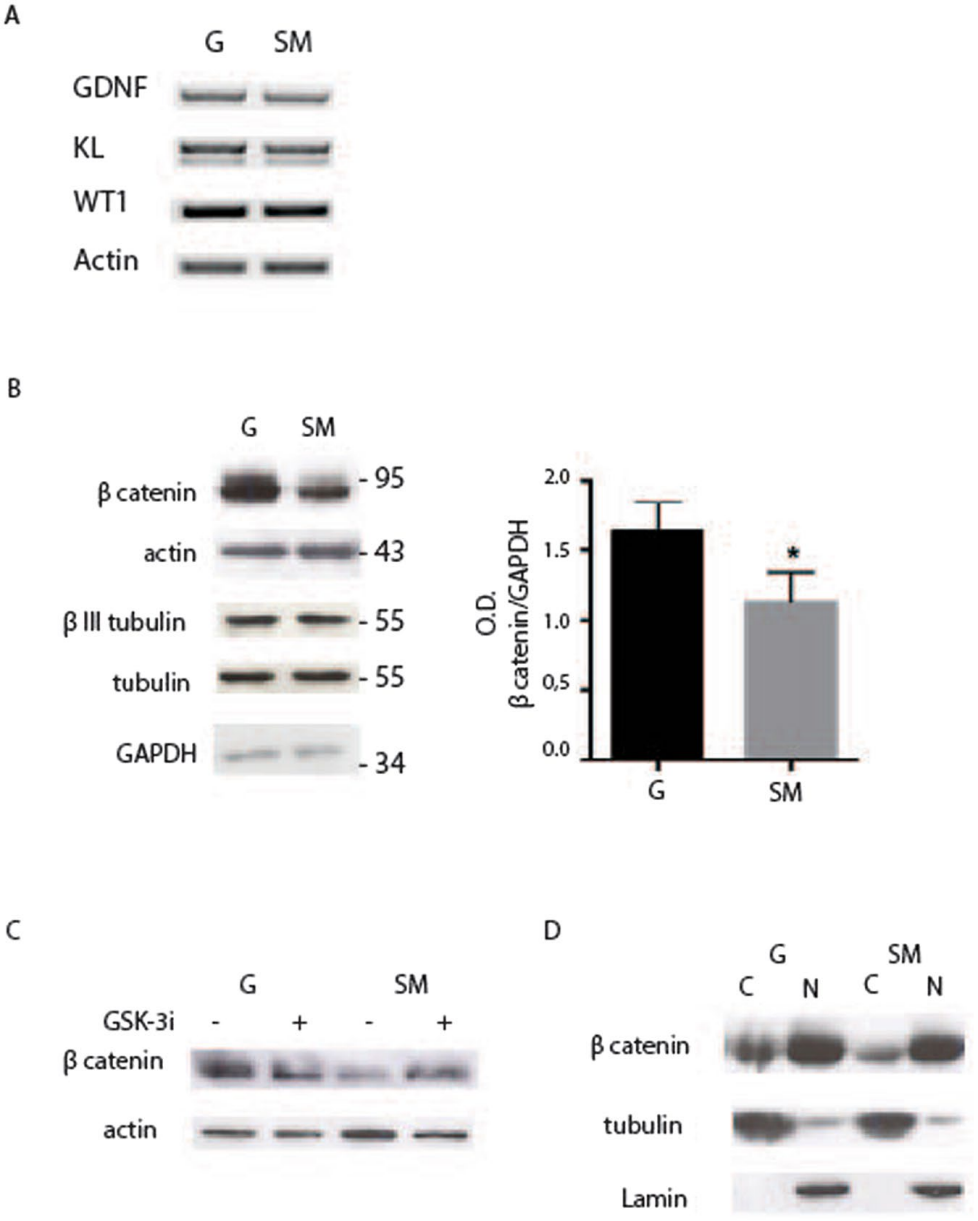
Microgravity causes a decrease in β-catenin protein expression. (**A**) Semiquantitative–PCR analysis of growth factors GDNF and KL in Sertoli cells cultured for 48 h at G or under SM. (**B**) Western blot analysis of cytoskeletal proteins in extracts from Sertoli cells cultured for 48 h at G or under SM. Densitometric analysis of β-catenin is reported on the right showing a decrease in the protein level under SM. (**C**) Western blot analysis of β-catenin in extracts from Sertoli cells cultured for 48 h at G or under SM, in the presence or not of GSK-3β inhibitor CHIR 99021. Treatment inhibits degradation of β-catenin caused by SM. (**D**) Western blot analysis of β-catenin distribution in the nuclear (N) and cytosolic (**C**) fractions of Sertoli cells cultured for 48 h at G or under SM. At least three different experiments were performed. Bars represent s.d. Asterisks indicate: *P < 0.05.

To understand the biological response of Sertoli cells to the different environmental stimuli induced by SM, we first analyzed, by semiquantitative RT-PCR, the expression of two trophic factors such as Glial-cell-line-derived neurotrophic factor (GDNF) and Kit ligand (KL), secreted by the Sertoli cells^1^. As shown in Fig. 3A they resulted unaffected by microgravity. Next we evaluated by western blot analysis the expression of the main cytoskeletal components. We show in Fig. 3B that no alterations were observed in the expression of tubulin, actin and βIII tubulin in Sertoli cells in RCCS with respect to G condition. However, we found that microgravity caused a significant decrease in β-catenin global expression (1.13 ± 0.18 at SM respect to 1.63 ± 0.21 at G) (Fig. 3B). In normal epithelial cells β-catenin is found associated at the cellular membrane at adherent junctions^22^, while any free cytoplasmic β-catenin is phosphorylated and targeted for ubiquination-dependent degradation by a protein complex formed by APC, GSK-3, CKIα, and Axin^23^. We observed that, by interfering with this process with the use of CHIR 99021, an inhibitor of GSK-3 β, the level of β-catenin in Sertoli cells cultured in RCCS could be restored up to that of cells cultured at G (Fig. 3C), indicating that microgravity induced a significant increase in β-catenin degradation. We next investigated the distribution of β-catenin between nuclear and cytoplasmic fraction. We show that β-catenin is present in the cytoplasm and is particularly concentrated in the nuclei and that microgravity reduced the cytoplasmic form but had no effect on the nuclear form (Fig. 3D). Our results indicate that SM did not induce significative alterations in the expression of two growth factors, KL and GDNF as well as in the expression of the main cytoskeleton components such as actin and tubulin, but caused a downregulation of cytoplasmic β-catenin protein level.

### SM affects aromatase expression in Sertoli cells

**Figure 4:**
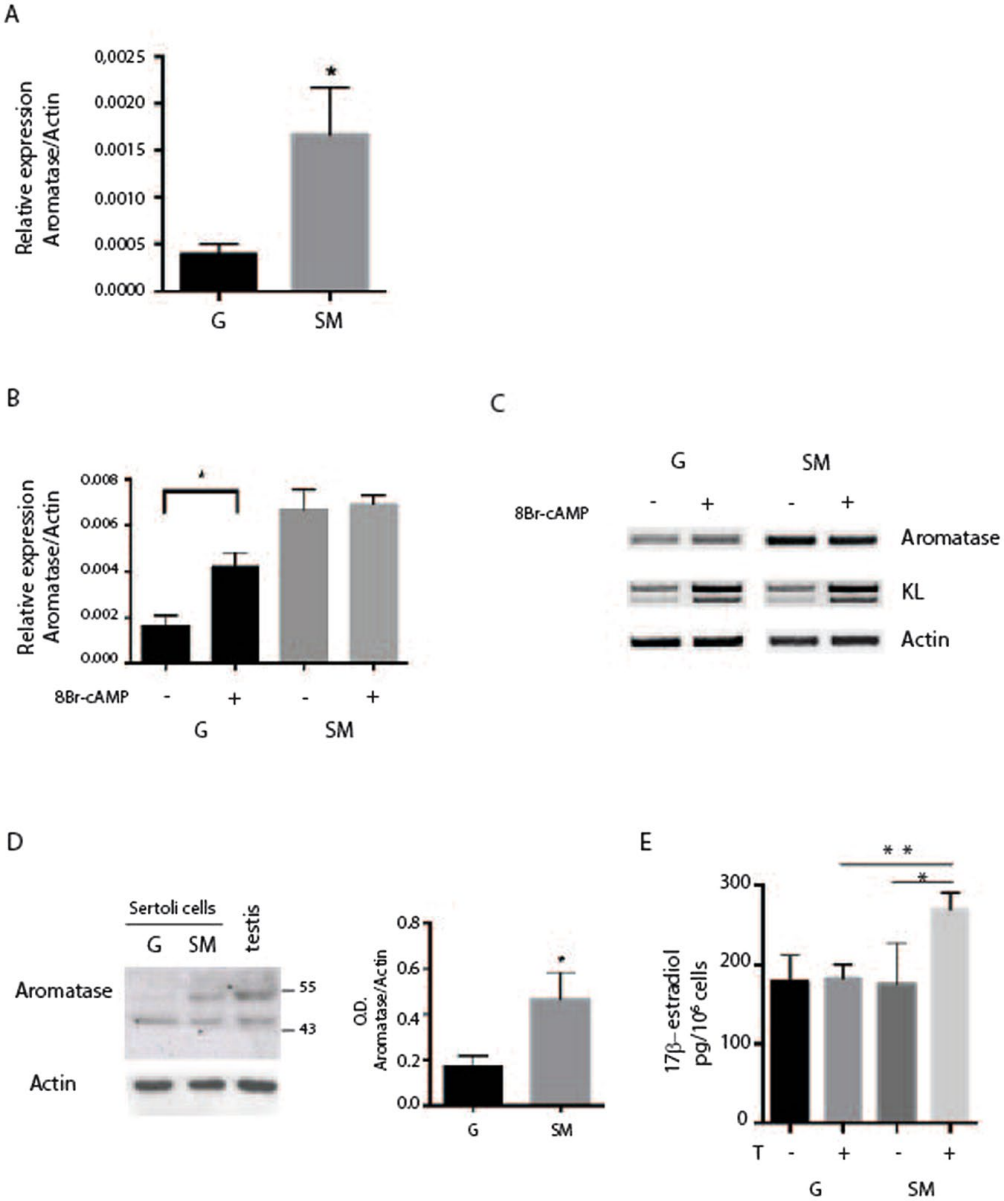
SM increases P450-aromatase expression in Sertoli cells. Sertoli cells were cultured for 48 h at G or in RCCS. (**A**) Real time PCR of P450-aromatase showing a strong increase of mRNA level under microgravity condition. (**B**) Real time-PCR for P450-aromatase in Sertoli cells treated or not with 1 mM 8-Br-cAMP. cAMP inducibility is lost under SM. (**C**) Semiquantitative RT-PCR of P450-aromatase and KL in Sertoli cells treated or not for 48 h with 1 mM 8-Br-cAMP. P450-aromatase expression is stimulated by 8-Br-cAMP at G but not under SM, while KL expression is stimulated by 8-Br-cAMP in both culture conditions. (**D**) Western blot analysis of P450-aromatase in Sertoli cells after 48 h of culture at G or under SM. Histogram on the right reports the mean of densitometric analysis of four different experiments. Values were normalized by reference to values for actin. (**E**) Chemiluminescence immunoassay for estrogen level in the culture medium of Sertoli cells. Sertoli cells were cultured in the presence or absence of 50 nM testosterone at G or in SM for 48 h. SM causes an increase of estrogen in the medium when cells are treated with testosterone. At least three different experiments were performed. Bars represent s.d. Asterisks indicate: *P < 0.05 and **P < 0.01.

In the testis of immature male rats, Sertoli cells represent primary source of estrogen, while in mature male, estrogen production in Sertoli cells strongly decreases^24^. We first aimed to evaluate, by real time PCR, the effect of microgravity on the expression of P450-aromatase in mouse prepuberal Sertoli cells. As shown in Fig. 4A, a highly significant increase of aromatase mRNA was detected in Sertoli cells cultured in RCCS, which resulted higher (0.0017 ± 0.001) with respect to culture at G (0.0004 ± 0.001). We also investigated the effects of SM on basal and 8-Br-cAMP stimulated aromatase mRNA levels by Real time-PCR. 8-Br-cAMP stimulated P450-aromatase expression in Sertoli cells cultured at G, but not in cells in RCCS, being the basal level of aromatase mRNA already high and therefore not substantially influenced by 8-Br-cAMP (Fig. 4B). On the other hand, semiquantitative PCR analysis of KL, whose expression is upregulated by 8-Br-cAMP at G, maintained this regulation also in SM (Fig. 4C). We next investigated the expression of aromatase at protein level by western blot analysis. The presence of an aromatase immunoreactive protein band of about 55 kDa was particularly faint in Sertoli cells at G but it was more readily detected under SM, indicating that microgravity caused an increase of aromatase expression (0.47 ± 0.11 at SM versus 0.173 ± 0.05 at G) also at protein level in primary mouse Sertoli cells (Fig. 4D).

Since aromatase abundance controls the levels of local estrogens, we investigated the presence of a functional P450-aromatase in Sertoli cells by measuring the amount of 17β-estradiol converted by androgen precursors (i.e. testosterone). Sertoli cells were cultured in the presence or absence of testosterone for 48 hours in RCCS or at G. At the end of treatment, the presence of 17β-estradiol was evaluated in the medium by chemiluminescence immunoassay. As shown in Fig. 4E, in absence of testosterone, the concentration of 17β-estradiol detected in the medium did not change between G and SM condition (178 pg/10^6^ cells ± 34 at G versus 175 pg/10^6^ cells ± 52 at SM). On the contrary, in the presence of testosterone we found a significant enrichment of 1,68 fold of 17β-estradiol secreted in the medium only under SM (160 pg/10^6^ cells ± 54 at G versus 268,7 pg/10^6^ cells ± 21,8 at SM) (Fig. 4D), in line with the increased expression of the enzyme under this condition.

## Discussion

Knowledge about the effects of microgravity on male reproduction is still in progress. Here we report the effects of SM on the expression of P450-aromatase in isolated mouse Sertoli cells. Studies on the effect of lack of gravity at the cellular level are limited. In mouse, microgravity has a direct effect on male germ cells; indeed it has been demonstrated that isolated mitotic spermatogonia cultured under SM in RCCS enter into meiosis in the absence of any added exogenous factor or contact with somatic cells^25^, while mouse pachytene spermatocytes undergo a spontaneous meiotic progression^26^. Here we established a 3D culture system of primary mouse Sertoli cells in RCCS and we compared some biological properties of these cells to those cultured in 3D at unit gravity. This approach allows to study the cell autonomous testicular cell responses to weightless not dependent on the influence of hormones. In both culture conditions, Sertoli cells formed cell aggregates that were similar in size. SM did not affect cell viability and apoptosis, as revealed by FACS analysis of sub G1 population, by Trypan blue staining and by western blot analysis of PARP-1 protein. Moreover, SM did not induce alterations in cell cycle and most of the cells were in G1 phase as those cultured at unit gravity. This result is in line with previous evidence indicating that most of Sertoli cells from prepuberal 17days old mice have already arrested their proliferative state^27, 28^. The expression of two important growth factors produced by Sertoli cells, GDNF and KL, was not altered under SM as well as the expression of the main cytoskeletal components. However, we showed that SM reduced the level of cytoplasmic β-catenin protein by promoting its degradation, since treatment of the cells with a specific GSK-3 β inhibitor, rescued the expression levels of the protein. Although the molecular mechanisms by which SM influences β-catenin expression remains unknown, we might hypothesize that the effect is linked to fluid shear stress to which cells are exposed in RCCS culture. In fact previous studies report that shear flow negatively regulated the level of β –catenin in colon cancer cells^29^. In these cells, shear stress is sensed and transduced by a pathway that involves laminin-5, α6β4 integrin, PI 3-kinase and Rac1 to selectively regulate the level of β-catenin, and future studies will be done to investigate this pathway also in Sertoli cells.

The most relevant finding of this study is that microgravity induced a very high upregulation of P450-aromatase expression at mRNA and protein level in Sertoli cells. We showed that in mouse, similarly to rat^11^, expression of P450-aromatase is very low in non-proliferating Sertoli cells, when they are cultured at unit gravity, but it was strongly upregulated under microgravity, with consequent enrichment in estradiol production. The high level of expression of aromatase under SM caused a loss of its cAMP inducibility. Indeed basal level of aromatase mRNA was high under SM and therefore not substantially influenced by 8-Br-cAMP. This effect is specific of this gene and not of other cAMP-regulated genes in Sertoli cells such as KL, suggesting that microgravity may modify the transcription of aromatase gene acting on transcription factors involved in its cAMP regulation. All together these results indicate that increased aromatase expression could affect the hormonal environment of testicular tissue under SM conditions, thus possibly affecting also circulating hormonal levels. Indeed, it has been already reported that male transgenic mice overexpressing aromatase in the testis showed estradiol levels higher than control mice, both locally and in the serum^30^.

Many studies established the importance of estrogens in male fertility^31–33^, as demonstrated by experimental models of mice lacking estrogen receptors or aromatase. Mice lacking ERα are sterile and sperms recovered from the cauda epididymis show low motility^33^, while aromatase deficient mice (ArKO) develop abnormal spermatogenesis with a blockage of germ cell maturation at the spermatid stage^34^. However, it should be underlined that estrogens may have an inhibitory influence of on Leydig cell steroidogenesis^35, 36^ and that high levels of this hormone have adverse effects on male reproduction^37, 38^. In accordance, increased estrogen levels have been observed in infertile men with low sperm production and quality^39^. The overexpression of aromatase in Sertoli cells under SM could also be associated with lower level of testosterone detected in rat and men after space flights and could be linked to increased aromatization of testosterone to estrogen^5, 6^. Moreover it may be speculated that microgravity could modify the expression of aromatase also in other tissues expressing the enzyme such ovarian granulosa cells, the placental syncytiotrophoblast, adipose and skin fibroblasts, bone, and brain. One consequence in men should be a change in T/E2 (Testosterone/17β-estradiol) balance, a parameter that is among the etiologic factors in idiopathic infertile males^40^. Beside the alterations in male fertility, higher levels of estrogen could have a role in promoting testicular tumors. It has been demonstrated that in mice, overexpression of aromatase results in increased estrogen production that leads to the induction of Leydig cell tumors^30^. The prolonged exposure to an excess of estrogens might have a more general role in inducing male reproductive tract malignancies such as testicular and prostatic tumors^41^. Interestingly, among somatic testis tumors, large cell calcifying Sertoli cell tumors^42^ and Sertoli cell tumors of Peutz-Jeghers syndrome have shown an enhanced expression of P450-aromatase^43, 44^.

In conclusion, the results from this study highlight the relationship between microgravity, P450-aromatase expression and estrogen production in Sertoli cells and suggest that long term weightless exposure of male astronauts may affect fertility and promote testicular cancers.

## Methods

### Cell culture

Primary Sertoli cell enriched-cultures from 17-days-old Swiss-CD1 mice were prepared as previously described^45^. Seminiferous tubules were prepared by sequential trypsin and collagenase digestion of dealbuginized testes. Tissue explanted was cultured at 32 °C in serum free minimum essential medium (MEM) supplemented with glutamine, nonessential amino acids, penicillin and streptomycin for 3 days and then they were treated with hypotonic solution (20 mM Tris-HCl, pH 7.5) to remove remaining germ cells. Cells were cultured at unit gravity in conventional tissue culture dishes with PBS 0.7% agarose, or at microgravity in a rotary cell culture system (RCCS) (Synthecon Inc., El Rio, Houston, TX, USA). In the RCCS condition 4 × 10^6^ Sertoli cells were seeded in disposable vessels with 10 ml MEM, using a rotation rate of 14 revolutions/min (RPM)^25^. Where indicated, Sertoli cells were cultured for 48 hours with 3 µM GSK3β inhibitor, CHIR 99021 (Axon Medchem)^46^ or with 1 mM 8-Br-cAMP (8-bromoadenosine 3′,5′-cyclic monophosphate, Sigma-Aldrich). All tested conditions were analyzed in triplicates. Animals were maintained and killed in accordance with European Community guidelines. Experimental protocols were performed in accordance with guidelines established by the European Legislation (Directive 2010/63/EU) and approved by University of Rome Tor Vergata IACUC and by Ministry of Health (legal authorization N. 140072016-PR).

### RT-PCR Analysis and quantitative Real Time PCR

Total RNA was extracted from Sertoli cells using Trizol reagent (Invitrogen) according to the manufacturer’s instructions and 1 μg was used for retrotranscription (RT) using M-MLV reverse transcriptase (Invitrogen). cDNA produced by the RT reaction was used as template for semiquantitative PCR analysis (GoTaq, Promega) or quantitative Real Time PCR performed using SSOADV Universal SYBR Green (BioRad) in a PRISM 7300 Sequence Detection System (Applied Biosystems). All the primers used are listed in Table 1.

**Table 1:** List of primers.

Aromatase	Fw: 5′-TCGGGCTACGTGGATGGT-3′ Rv: 5′-GAGCTTGCCAGGCGTTAAAGT-3′
GDNF	Fw: 5′-GGAGTTAATGTCCAACTGGG-3′ Rv: 5′-TACATCCACACCGTTTAGCG-3′
Kit Ligand	Fw: 5′-CTCCGAAGAGGGCCAGAAACTGAT-3′ Rv: 5′-CACAATTACACCTCTTGAAATTCTCT-3′
WT-1	Fw: 5′-CCAAATGACCTCCCAGCTTGAATG-3′ RV: 5′-TTCTGACAACTGTGCCACCACAG-3′
Actin	Fw: 5′-CTGTCGAGTCGCGTCCAC-3′ Rv: 5′-GCTTTGCACATGCCGGAG-3′

### Western blotting

For western blotting analysis, cells were lysed in 1% Triton X-100, 150 mM NaCl, 15 mM MgCl_2_, 15 mM EGTA, 10% Glycerol, 50 mM Hepes (pH 7.4) with protease inhibitors. Proteins were separated by SDS–10% polyacrylamide gel electrophoresis and transferred to polyvinylidene fluoride (PVDF) membrane (Amersham). For cellular protein fractionation, Sertoli cells were harvested and lysed in buffer A (25 nM HEPES pH 7.9, 150 mM NaCl, 10 nM EDTA pH 8, 0.1% NP40, 50 mM β-glycerophosphate with protease inhibitor mix). The homogenates were centrifuged for 10 min at 3,000 rpm to pellet nuclei. The supernatants were collected and used as cytosolic fractions. Nuclear fractions were extracted with buffer B (50 mM Tris/HCl pH 7.5, 150 mM NaCl, 0.25% sodium deoxycholate 10 μg/ml phenylmethylsulfonyl fluoride and protease inhibitor mix), followed by centrifugation for 15 min at 13000 rpm. Equal cellular amounts of each fraction were used for Western blot analysis. The membrane was blocked in TBS–5% skim milk powder for 1 h. The following primary antibodies (1:1000) were used: rabbit anti-actin (a2066, Sigma-Aldrich), rabbit anti-β catenin (c2206, Sigma-Aldrich), rabbit anti-PARP (sc8007, Sigma-Aldrich), mouse anti- βIII tubulin (T8578, Sigma-Aldrich), mouse anti-tubulin (sc5286, Santa-Cruz), rabbit anti-lamin B1 (sc-30264, Santa-Cruz), rabbit anti-GAPDH (sc-32233, Santa Cruz), rabbit anti-P450-aromatase (sc14244, Santa Cruz). Incubation of the membrane with the primary antibody was carried out at 4 °C overnight in PBS–5% BSA and then appropriate horseradish peroxidase-conjugated secondary antibodies (Jackson ImmunoResearch) were used for detection with the ECL and ECL-plus systems (Amersham Biosciences) and visualized by chemiluminescence.

### Immunohistochemistry

Cell spheroids were harvested and fixed in 4% buffered-formalin. Sections were pre-treated for 10 min with heat-induced epitope retrieval in buffer pH 9.00 (EnVisionTM FLEX Target Retrieval Solution High pH, Dako Denmark A/S, Glostrup, Denmark). Slides were then incubated for 20 min at room temperature with anti Wilms’ Tumor (WT1) (Dako) mouse monoclonal antibody clone 6F-H2 at 1:100 dilution. Staining procedures were performed by EnvisionTM FLEX + (Dako Denmark A/S) Detection System and AutostainerLink 48 instrument following dealer’s instructions.

Sections were countrastained with H&E, mounted with Permount (Fisher) and analyzed with a Zeiss Axioskope microscope.

### FACS (Fluorescence Activated Cell Sorter)

Cell cycle was analyzed on disaggregated Sertoli cells. Cells were fixed in 1% paraformaldehyde for 30 min, washed in PBS and incubated for 16 h with 70% ethanol. Cells were stained with propidium iodide (10 μg/ml) and analyzed on a FACS Calibur Flow Cytometer (Becton Dickinson, San Jose, CA).

According to the manual of BrdU (Bromodeoxyuridine) labeling/detection kit (Roche, Nutley, NJ), 10 µM BrdU labeling medium was added to the Sertoli cultures and allowed to incubate overnight at 32 °C under 5% CO_2_. Afterwards, cells were processed for FACS analysis. Cells were washed two times in Washing buffer (PBS 0.5% Tween 20), fixed overnight with Ethanol 70% and stained with anti-Brdu (BD Biosciences, San Jose, CA, diluted 1∶50) according to the manufacturer’s instruction. After two washing in PBS, 0.5% Tween 20, cells were incubated with an anti-mouse FITC-conjugated secondary antibody (Alexa Fluor® 488 Dye 1∶350) at 4 °C for 45 min in the dark.

### Hormone dosage

Sertoli cells were cultured in the presence or absence of 50 nM testosterone (Sigma Aldrich) for 48 hours at G or under SM. At the end, the levels of 17β-estradiol (E2) in the culture medium were measured by an automated chemiluminescence immunoassay direct system (ADVIA Centaur XP, Siemens technology, USA) The kit is able to detect steroid contents (E2 ≥ 10 pg/mL).

### Statistical Analysis

Statistical analysis was obtained performing at least three different experiments. Continuous variables were summarized as means ± s.d. A significance value threshold of 0.05 was used for the current analysis. Student’s t-test was used to test for differences between two independent groups, whereas one-way ANOVA was used to test for differences among three or more independent groups. All statistical tests were carried out using the GraphPad Prism statistical analysis software package, version 6.0.
